# Evaluation of JNJ-54717793 a Novel Brain Penetrant Selective Orexin 1 Receptor Antagonist in Two Rat Models of Panic Attack Provocation

**DOI:** 10.3389/fphar.2017.00357

**Published:** 2017-06-09

**Authors:** Pascal Bonaventure, Christine Dugovic, Brock Shireman, Cathy Preville, Sujin Yun, Brian Lord, Diane Nepomuceno, Michelle Wennerholm, Timothy Lovenberg, Nicolas Carruthers, Stephanie D. Fitz, Anantha Shekhar, Philip L. Johnson

**Affiliations:** ^1^Janssen Research & Development, LLC, San DiegoCA, United States; ^2^Department of Psychiatry, Indiana University School of Medicine, IndianapolisIN, United States; ^3^Stark Neurosciences Research Institute, Indiana University School of Medicine, IndianapolisIN, United States; ^4^Department of Anatomy and Cell Biology, Indiana University School of Medicine, IndianapolisIN, United States

**Keywords:** orexin, hypocretin, panic, anxiety, hypercapnia

## Abstract

Orexin neurons originating in the perifornical and lateral hypothalamic area are highly reactive to anxiogenic stimuli and have strong projections to anxiety and panic-associated circuitry. Recent studies support a role for the orexin system and in particular the orexin 1 receptor (OX1R) in coordinating an integrative stress response. However, no selective OX1R antagonist has been systematically tested in two preclinical models of using panicogenic stimuli that induce panic attack in the majority of people with panic disorder, namely an acute hypercapnia-panic provocation model and a model involving chronic inhibition of GABA synthesis in the perifornical hypothalamic area followed by intravenous sodium lactate infusion. Here we report on a novel brain penetrant, selective and high affinity OX1R antagonist JNJ-54717793 (1S,2R,4R)-7-([(3-fluoro-2-pyrimidin-2-ylphenyl)carbonyl]-*N*-[5-(trifluoromethyl)pyrazin-2-yl]-7-azabicyclo[2.2.1]heptan-2-amine). JNJ-54717793 is a high affinity/potent OX1R antagonist and has an excellent selectivity profile including 50 fold versus the OX2R. *Ex vivo* receptor binding studies demonstrated that after oral administration JNJ-54717793 crossed the blood brain barrier and occupied OX1Rs in the rat brain. While JNJ-54717793 had minimal effect on spontaneous sleep in rats and in wild-type mice, its administration in OX2R knockout mice, selectively promoted rapid eye movement sleep, demonstrating target engagement and specific OX1R blockade. JNJ-54717793 attenuated CO_2_ and sodium lactate induced panic-like behaviors and cardiovascular responses without altering baseline locomotor or autonomic activity. These data confirm that selective OX1R antagonism may represent a novel approach of treating anxiety disorders, with no apparent sedative effects.

## Introduction

Orexin neurons are located in the perifornical and lateral hypothalamus of rodents (Peyron et al., 1998), and humans (Thannickal et al., 2007). The role of orexins (OX-A and OX-B) and orexin receptors (OX1R and OX2R) in complex emotional behavior including innate anxiety and panic and fear associated learning is emerging (Johnson et al., 2010; Boss and Roch, 2015; Flores et al., 2015). For instance, optogenetically stimulating orexin neurons in rats increases anxiety-like states in anxiety-related neural circuits (Heydendael et al., 2013) as well as stress hormone release and tachycardia (Bonnavion et al., 2015). In addition, artificially increasing orexin-A levels in the cerebrospinal fluid of rodents increases anxiety associated behaviors (Suzuki et al., 2005), which is consistent with elevated orexin levels being associated with increases in anxiety symptoms in neuropsychiatric patients (Johnson et al., 2010). Severe anxiety such as panic attacks are also more prevalent during the wake phase (de Beurs et al., 1994) when orexin levels are at their highest levels in humans (Mignot et al., 2002) and in rats (Desarnaud et al., 2004).

**FIGURE 1:**
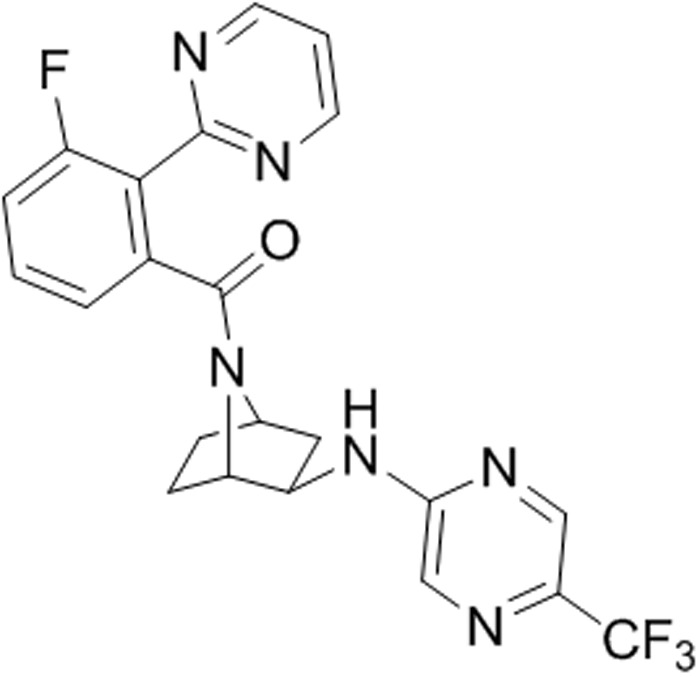
Chemical structure of JNJ-54717793 (1S,2R,4R)-7-([(3-fluoro-2-pyrimidin-2-ylphenyl)carbonyl]-*N*-[5-(trifluoromethyl)pyrazin-2-yl]-7-azabicyclo[2.2.1]heptan-2-amine).

Excitation of the perifornical hypothalamic area, which contains particularly high concentration of orexin neurons, produces robust flight and escape behaviors, and cardioexcitation in rodents (Shekhar and DiMicco, 1987; Anderson and DiMicco, 1990; Shekhar et al., 1990; Soltis and DiMicco, 1992; Samuels et al., 2002), and induces core symptoms of panic attacks in humans [e.g., fear of dying, cardiorespiratory symptoms, and thermal sensations (Rasche et al., 2006; Wilent et al., 2010, 2011)]. In orexin knockout mice, cardiovascular responses are diminished following perifornical hypothalamic area stimulation (Kuwaki et al., 2008). Panic disorder is characterized by recurrent spontaneous panic attacks, which can be reliably induced with interoceptive stimuli such as intravenous 0.5 M sodium lactate and 5 min of 5–7% hypercapnic gas exposure, which do not induce panic attacks in healthy controls (Pitts and McClure, 1967; Woods et al., 1988; Gorman et al., 1994). Exposing healthy humans to higher concentrations of hypercapnic gas (i.e., 20% CO_2_ inhalation) will also induce core features of panic attacks (Forsyth et al., 2000). Collectively, these two panic paradigms can be modeled in rats: (1) chronic disinhibition of the perifornical hypothalamic area orexin region also produces rats that are vulnerable to displaying panic-like responses to interoceptive stimuli, such as sodium lactate (see review, Johnson and Shekhar, 2012); and (2) exposing naïve rats to 20% hypercapnic gas exposure also induces panic-associated responses. Both panic models, using stimuli that signal a threatening internal body state change, activate OX neurons in the perifornical hypothalamic area (Williams et al., 2007; Johnson et al., 2010, 2012c,d) and increase panic associated behaviors (e.g., flight like locomotion and anxiety in a social interaction (SI) test and defensive burying test) and panic associated dyspnea (Hickman et al., 2016) and cardioexcitation (Johnson et al., 2010, 2012c,d, 2015). In these panic models, silencing prepro orexin (sodium lactate only) or selective OX1R antagonists attenuate panic-like responses to CO_2_ and sodium lactate (Johnson et al., 2010, 2012c, 2015). Additionally, in rats a selective OX1R antagonist blocks cellular responses in panic and anxiety brain circuits (e.g., amygdala and dorsal periaqueductal gray and sympathetic nuclei) post-administration of a panicogenic drug (Johnson et al., 2012c), and in humans recent imaging data indicate that a selective OX1R antagonist produced a region-dependent inhibition of yohimbine-induced activation in fronto-hippocampal regions as well as in several key components of the extended amygdala (Gozzi et al., 2013). The OX1R inhibition in the amygdala is consistent with preclinical studies demonstrating that the OX system is implicated in amygdala regulation of fear-associated learning (Johnson et al., 2012a; Sears et al., 2013; Flores et al., 2014). Overall, there is compelling evidence that overactivation of the OX1R pathway is associated with hyper-excited or hyper-active states; thus conceptually, a selective OX1R antagonist might normalize overexcited networks without inducing sedation (Bonaventure et al., 2015b). Based on these preclinical data, we developed a selective OX1R antagonist with suitable drug-like properties. A separate paper describes the new OX1R antagonist JNJ-54717793 (**Figure 1**) synthesis, structure activity relationship, pharmacokinetics in preclinical species (Shireman et al., submitted).

Here, we report a comprehensive pharmacological characterization of JNJ-54717793 and its evaluation in two well-established models of panic provocation. *In vitro* affinity and potency for the human and rat OX1R were determined by radioligand binding and *in vitro* functional assays. *In vivo* target engagement was measured after oral dosing of JNJ-54717793 in rat brain using *ex vivo* receptor occupancy. We have recently shown that while selective OX1R antagonism did not affect sleep-wake states, additional pharmacological blockade of OX1R to OX2R inhibition elicited a disinhibition of rapid eye movement (REM) in rats and mice (Dugovic et al., 2014). Therefore, the effect of the selective OX1R antagonist on sleep was tested in OX2R knockout (KO) mice to demonstrate *in vivo* functional target engagement.

## Materials and Methods

### Chemicals

Almorexant, EMPA, GSK-1059865, SB-674042, and JNJ-54717793 were synthetized at Janssen Research & Development, LLC. Peptides were obtained from Bachem (Torrance, CA, United States).

### Animals

All animal procedures performed in this study were in accordance with the Guide for Care and Use of Laboratory Animals adopted by the US National Institutes of Health (NIH Publication no. 80-23 revised 1996) and the guidelines of the Institutional Animal Care and Use Committee. Animals were housed under controlled conditions with a 12/12 h light/dark schedule and temperature of 22 ± 2°C. Food and water were provided *ad libitum*. Experiments were performed after animals had acclimated for at least 1 week unless stated otherwise.

#### *In Vitro* Radioligand Binding Assays

Human or rat OX1R binding was measured in competitive radioligand binding assays using [^3^H]SB-674042 (1-(5-(2-fluoro-phenyl)-2-methyl-thiazol-4-yl)-1-((*S*)-2-(5-phenyl-(1,3,4)oxadiazol-2-ylmethyl)-pyrrolidin-l-yl)-methanone) as a tracer (4 nM, specific activity 35 Ci/mmol) as previously described (Langmead et al., 2004). Cell membranes were prepared from clonal CHO K1 cells transfected with the human OX1R or clonal HEK-293 cells transfected with the rat OX1R. Dilutions of test compounds were made in Dulbecco’s PBS from 10 mM stocks dissolved in dimethyl sulfoxide. After a 60 min incubation at room temperature, binding reactions were filtered. The membranes were counted in a scintillation counter. Non-specific binding was determined in the presence of 10 μM almorexant. Affinities of compounds for the human OX2R were measured by competitive radioligand binding using tritiated *N*-ethyl-2-[(6-methoxy-pyridin-3-yl)-(toluene-2-sulfonyl)-amino]-*N*-pyridin-3-ylmethyl-acetamide (EMPA) (2 nM, specific activity 27 Ci/mmol) (Malherbe et al., 2009). Cell membranes were prepared from a stable pool of HEK-293 cells transfected with the human OX2R. Non-specific binding was determined with 10 μM almorexant.

The K_i_ of the test compounds was calculated based on non-linear regression (one site competition) using Graphpad Prism.

The selectivity of JNJ-54717793 was evaluated in a large variety of ion-channels, transporters and receptor-binding assays. These assays were performed by Eurofins (Celles L’Evescault, France).

#### *In Vitro* Functional Assays (Calcium Mobilization Assays)

Stably transfected CHO-K1 cells for the human OX1R or HEK-293 cells for the rat OX1R were used for the *in vitro* functional assays. The human OX2R functional assay used PFSK-1 cells which are a human neuroectodermal cell line that innately expresses the OX2R. Since the intracellular calcium response is transient and not consistent with equilibrium assumptions, the assays were performed by giving a standard, EC_80_ dose of the OX agonist and calculating a pK_B_ from inhibition of the agonist response by a dose range of the antagonists. The cells were plated in black 96 well tissue culture plates with clear bottoms at 50,000 cells/well and grown overnight at 37°C in 5% carbon dioxide (CO_2_). Dilutions of the antagonist were prepared in Hanks Balanced Salt Solution (HBSS) from 10 mM DMSO stocks, while dilutions of OX peptides (OX-A for OX1R assays, OX-B for OX2R assays) were prepared in HBSS + 0.1% bovine serum albumin. On the day of the assay, a 2X dye-loading solution (BD Calcium Assay Kit) was added to the cells and incubated for 45 min at 37°C in 5% CO_2_. Dilutions of the test compounds were added and the cells were incubated at room temperature for 15 min. The cell plate was then transferred to the Molecular Devices Fluorometric Imaging Plate Reader (FLIPR) Tetra instrument, which adds the OX agonist and monitors changes in fluorescence which reflect intracellular calcium levels.

Results were calculated using GraphPad Prism (San Diego, CA, United States) software. Raw data from the FLIPR Tetra was exported as the difference between maximum and minimum fluorescence observed for each well. A non-linear regression was used to determine the agonist EC_50_ and antagonist IC_50_ for each plate, then the antagonist K_B_ was calculated according to Cheng and Prusoff (Cheng and Prusoff, 1973).

#### *Ex Vivo* Receptor Occupancy Assay

Experiments were performed as previously described (Dugovic et al., 2009) in male Sprague-Dawley rats (300–400 g, Charles River Laboratories, San Diego, CA, United States). The animals were euthanized using carbon dioxide and decapitated at different time points after drug administration (*n* = 3 per time point or dose regimen). Brains were rapidly frozen on powdered dry ice and stored at -80°C before sectioning. Plasma samples were also collected for bioanalysis (LC–MS/MS). Twenty micron thick tissue sections at the level of the tenia tecta were prepared for autoradiography. OX1R radioligand binding autoradiography was determined at room temperature with 5 nM [^3^H]SB-674042. Sections were incubated for 10 min to minimize dissociation. Non-specific binding was determined in the presence of 10 μM SB-674042. *Ex vivo* receptor labeling was expressed as the percentage of receptor labeling in corresponding brain areas (i.e., tenia tecta) of vehicle-treated animals. The percentage of receptor occupancy was plotted against time or dosage using GraphPad Prism (GraphPad Software, San Diego, CA, United States). Percentage of receptor occupancy was also plotted against drug plasma or brain concentration. Pharmacokinetic parameters were analyzed using a non-compartmental model using the software package WinNonlin Version 4.0.1 (Pharsight, Palo Alto, CA, United States).

#### Sleep Recording and Analysis in Rats and Mice

Sleep experiments were conducted in male Sprague-Dawley rats (350–450 g, Harlan Laboratories, Livermore, CA, United States) and in male C57Bl6 OX2R knockout (KO) and corresponding wild-type mice (30–35 g, Charles River Laboratories, San Diego, CA, United States) as described previously (Dugovic et al., 2009). Animals were chronically implanted with telemetric devices (Data Sciences International, St. Paul, MN, United States) for the recording of electroencephalogram (EEG) and electromyogram (EMG) signals. Polysomnographic waveforms were analyzed per 10-s epoch and classified as wake, non-rapid eye movement (NREM) or REM sleep by using the computer software program SleepSign (Kissei Comtec, Nagano, Japan). For each experiment, EEG and EMG signals were recorded for up to 6 h after administration of the tested compounds. Analysis of sleep parameters included latency to NREM sleep (defined as the time interval to the first six consecutive NREM epochs) and REM sleep (the first two consecutive REM epochs post-treatment), and the duration of NREM and REM.

Results were averaged and expressed as mean ± SEM in defined time intervals. To determine whether differences were significant at a given interval, either paired Student’s *t*-test or two-way analysis of variance (ANOVA) (interaction time × treatment), repeated measures followed by Bonferroni *post hoc* test was performed.

#### Normoxic, 20% CO_2_ Panic Provocation Model

Experiments were performed in male Sprague-Dawley rats (300–350 g, Harlan Laboratory, Indianapolis, IN, United States). Using CO_2_ (ProCO_2_) and O_2_ (ProO_2_) sensors in our enclosed flow cages (12 inch width × 12 inch height × 24 inch length), we have previously verified that O_2_ and CO_2_ concentrations remain normal with atmospheric air infusion, and that only the CO_2_ concentrations rapidly increase from <1 to 20% at the 5 min time point for CO_2_ challenge (Johnson et al., 2005). In a counter-balanced design (i.e., all rats received each drug treatment with at least 48 h between treatments), rats were systemically treated with a control vehicle or a selective OX1R antagonist, and then placed into the chamber where atmospheric air was being infused. All rats had infusions of the following: (1) 5 min infusion of atmospheric gas (<1% CO_2_, 21% O_2_, 79% N_2_: Praxair, Inc.) for baseline measurements (∼45–60 min post-treatment), then (2) either the control gas or experimental normoxic, hypercarbic gas (20% CO_2_, 21% O_2_, 59% N_2_: Praxair) for 5 min (note: for control rats the atmospheric gas was turned off and back on again at the beginning and end of this infusion to be identical to the manipulations for the hypercarbic gas challenge), and finally (3) 5 min infusion of atmospheric gas. Following exposure to hypercarbic and atmospheric air gasses, rats were immediately placed in the open-field box for a 5 min period then assessed in SI test for 5 min. The data reported are changes in activity, expressed in 1 min bins, relative to the average of the baseline measurement (t-5 to t-1 minute) from each rat.

#### Sodium Lactate Panic Provocation Model

Experiments were performed in male Sprague-Dawley rats (300–350 g, Harlan Laboratory, Indianapolis, IN, United States). Prior to and during surgeries, rats were anesthetized with a nose cone connected to an isoflurane system (MGX Research Machine; Vetamic, Rossville, IN, United States). Radiotelemetry probes [Cat. no. HD-S11, Data Sciences International, St. Paul, MN, United States] were surgically implanted into the peritoneal cavity and sutured to the muscle wall in order to assess general motor activity. A pressure transducer was implanted into the femoral artery to assess cardiovascular responses [i.e., mean arterial blood pressure (MAP) and heart rate (HR)]. Rats were also fitted with femoral venous catheters for 0.5 M i.v. sodium lactate (NaLac) infusions, as previously described (Shekhar et al., 1996). After 3–5 days of recovery, rats were anesthetized and 26 gauge T-shaped cannulae (Cat. no. 3260PG, Plastics One, Inc., Roanoake, VA, United States) were directed at cardio-excitatory Perifornical hypothalamic area regions [(Shekhar and Keim, 1997) bregma: 1.2 mm posterior, +2.1 mm lateral, +9.1 mm ventral and adjusted for approaching at a 10° angle toward the midline with the stereotaxic incisor bar elevated 5 mm above the interaural line] and cemented into place. The 22 gauge side arm was then attached, via PE-60 tubing, to an osmotic minipump (prefilled with 1-allyglycine solution chronically infused at 3.5 nmol/0.5 μl per hour) and sutured into place subcutaneously at the nape of the neck (DURECT Corporation, Model no. 2002). Previous studies have determined that the dose of 1-allyglycine utilized here reduces local GABA concentrations by approximately 60% following unilateral infusions (Shekhar and DiMicco, 1987; Abshire et al., 1988; Shekhar et al., 1996, 2006; Shekhar and Keim, 1997).

Five days following 1-allyglycine infusion onset, in a counterbalanced design and with 48 h between crossover, rats were orally dosed with either JNJ-54717793 at 3, 10, or 30 mg/kg, or vehicle as a control group 60 min prior to the 15 min 0.5 M sodium lactate challenge. DSI DATAQUEST software was used to monitor and record mean arterial pressure, HR, and motor activity which were recorded continuously in freely moving conscious rats and are expressed as a 20 min time course. The data reported are changes in mean arterial blood pressure, HR, and activity from the average of the baseline (t-5 to t-1) from each rat.

Baseline SI testing was done 7–8 days following radiotelemetry surgery recovery, and repeated again 2–3 days later during drug treatment crossover. On experimental drug testing days, the SI test was performed 5 min after the offset of the sodium lactate challenge with different partners each time. The SI box dimension were 0.9 m long × 0.9 m wide × 0.3 m height. The SI test is a validated test of experimental anxiety-like behavior in rats that is sensitive to FDA approved treatments for anxiety disorder symptom management that includes benzodiazepines and selective serotonin reuptake inhibitors (SSRIs) (Sanders and Shekhar, 1995; Shekhar and Katner, 1995). All behavioral tests were digitally videorecorded with a camera above the box. The “experimental” rat and an unfamiliar “partner” rat were both allowed to individually habituate to the box for a 5 min period 24 h prior to each SI test. During the SI test, the two rats were placed together in the center of the box, and the total duration (sec) of non-aggressive physical contact (grooming, sniffing, crawling over and under, etc.) initiated by the “experimental” rat is quantified over a 5 min duration. A baseline SI test was performed at least 72 h after i.v. catheterization, but prior to osmotic minipump implantation. Another SI test was performed 5 days following minipump infusions and immediately following saline or sodium lactate infusions. Video recorded sessions were scored at a later time by Stephanie D Fitz, whom was blind to any drug treatment.

Following experiments, all rats were anesthetized and decapitated, their brains were removed, frozen, sectioned (30 μm) and stained with Neutral Red for determination of injection cannulae placements.

Each dependent variable for assessment of behavior and radiotelemetry data was respectively analyzed using a one way ANOVA, or a one way ANOVA with repeated measures with *drug treatment* as the main factor and time as repeated measures. In the presence of significant main effects *post hoc* tests were conducted using a parametric Fisher’s LSD test. Within subjects time effects were also assessed using a Dunnett’s one way analysis with the minute prior to the i.v infusion used as the control. Statistical significance was accepted with *p* < 0.05. All statistical analyses were carried out using SPSS 13.0 (SPSS, Inc., Chicago, IL, United States) and all graphs were generated using SigmaPlot 2001 (SPSS, Inc., Chicago, IL, United States) or Graphpad Prizm 7 Software, Inc. for Windows.

## Results

### JNJ-54717793 is a Selective High Affinity OX1R Antagonist

The affinity of JNJ-54717793 for the human and rat OX1R was determined by competitive radioligand binding using [^3^H]-SB674042. To determine the selectivity ratio versus the OX2R, the affinity of the compounds for the human OX2R was determined by competitive radioligand binding using [^3^H]-EMPA as the radioligand.

JNJ-54717793 showed high affinity binding to the human and rat OX1R, with pK_i_ values of 7.83 and 7.84 nM, respectively (**Table 1**). The binding selectivity of JNJ-54717793 at the human OX1R compared to the human OX2R was substantial (∼50 fold).

**Table 1:** *In vitro* binding affinities (pK_i_) and *in vitro* functional potencies (pK_b_) of JNJ-54717793 at the human and rat OX1R and OX2R.

	Affinity (pK_i_)	Potency (pK_b_)
hOX1R	7.83 ± 0.16 (12)	7.78 ± 0.31 (5)
rOX1R	7.84 ± 0.12 (17)	7.45 ± 0.20 (2)
hOX2R	6.14 ± 0.14 (16)	6.51 ± 0.24 (4)

*The values are expressed as average ± SEM, and the number of triplicate experiments is indicated between parentheses.*

JNJ-54717793 was assayed by binding in a panel of 50 receptors, ion channels and transporters assays including adenosine (A_1_, A_2A_, A_3_), adrenergic (α_1_, α_2_, α_1_), angiotensin (AT_1_), dopamine (D_1_, D_2_), bradykinin (B_2_), cholecystokinin (CCK_A_), galanin (GAL_2_), melatonin ML_1_), muscarinic (M_1_, M_2_, M_3_), neurotensin (NT_1_), neurokinin (NK_2_, NK_3_), opiate (μ, κ, δ), serotonin (5-HT_1A_, 5-HT_1B_, 5-HT_2A_, 5-HT_3_, 5-HT_5A_, 5-HT_6_, 5-HT_7_), somatostatin, vasopressin (V_1a_), norepinephrine transporter, dopamine transporter and ion channels (sodium, calcium, potassium, and chloride). JNJ-54717793 at concentrations up to 10 μM had no significant affinity for any receptor/transporter/ion channel (<50% inhibition at 10 μM) other than the OX1R.

The functional antagonism of JNJ-54717793 for the human or rat OX1R was determined by measuring changes in intracellular calcium in cell culture assays in response to an EC_80_ dose of OX-A. Functional antagonism data, summarized in **Table 1**, showed that the high affinity OX1R binding of JNJ-54717793 was reflected in potent functional activity. The pK_B_ values correlated well with the pK_i_ values for the human and rat OX1R. The binding selectivity of JNJ-54717793 at the OX1R compared to the OX2R was confirmed at the functional level (**Table 1**).

### JNJ-54717793 Crosses the Blood Brain Barrier and Occupies the OX1R in Rat Brain after Systemic Administration

**FIGURE 2:**
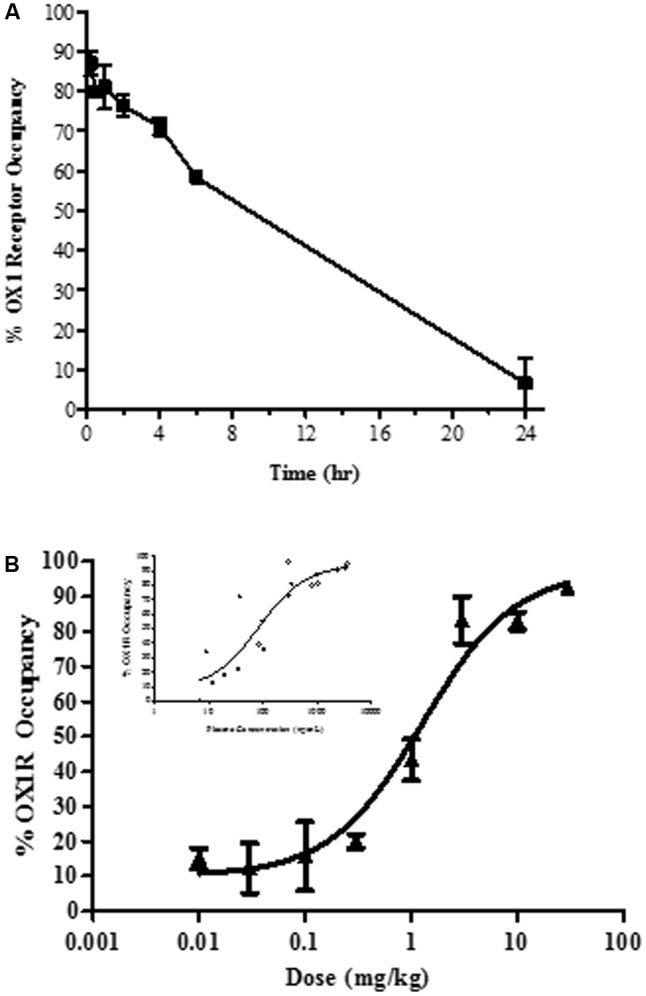
*Ex vivo* brain OX1R binding autoradiography by JNJ-54717793 measured in the tenia tecta **(A)** Duration of occupancy for JNJ-54717793 post-oral administration in rats (30 mg/kg). **(B)** Occupancy versus dose measured at 1 h. Insert graph showed occupancy versus plasma exposure. Results are expressed as average percentage receptor occupancy versus vehicle treated rats ± SEM (*n* = 3).

*In vivo* occupancy of the OX1R was assessed by *ex vivo* receptor binding autoradiography of [^3^H]SB-674042 in rat brain tissue sections at the level of the tenia tecta. Time-dependency and dose-dependency were assessed after oral dosing (**Figure 2**).

Oral administration of JNJ-54717793 inhibited [^3^H]SB-674042 binding to the rat tenia tecta, indicating sufficient oral bioavailability and brain penetration. After acute oral administration of 30 mg/kg, maximal OX1R occupancy was observed at 15 min (87 ± 3%, **Figure 2A**) corresponding to a plasma concentration of 3733 ± 440 ng/ml. The level of OX1R occupancy remained above 58% for the first 6 h and then drop to negligible level of occupancy.

For dose-dependency, *ex vivo* receptor occupancy was measured 60 min after drug treatment (close to the T_max_, observed in a rat pharmacokinetic study, result not shown). The plateau for maximal OX1R occupancy (∼80 to 90%) was reached at 3 mg/kg (**Figure 2B**) corresponding to a plasma concentration of ∼310.6 ± 23.5 ng/ml. The ED_50_ was measured at 1.3 mg/kg (corresponding to a plasma exposure of 84 ng/ml, 95% confidence interval 26 to 277 ng/ml).

*In vivo* OX1R occupancy was also determined at various time points following subcutaneous dosing of 10 mg/kg of JNJ-54717793 (result not shown). Maximal OX1R occupancy (83 ± 7%) was observed at 30 min. The plasma concentration for maximal receptor occupancy was determined to be 2211 ± 339 ng/ml. At the 2 h time point, JNJ-54717793 still displayed 65% OX1R occupancy.

### JNJ-54717793 Minimally Affects Spontaneous Sleep in Rats

Rats were subcutaneously injected with the OX1R antagonist JNJ-54717793 (10 mg/kg) at 2 h into the light phase. There were no significant differences between JNJ-54717793 and vehicle conditions (paired Student’s *t*-test) in most of the sleep parameters examined over the 2 h period following the treatment. Specifically, NREM sleep latency and duration were not altered. REM sleep latency was slightly but significantly reduced without producing a significant impact on REM sleep duration (**Table 2**).

**Table 2:** Effects of the OX1R antagonist JNJ-54717793 on sleep parameters in rats.

	NREM latency	REM latency	NREM duration	REM duration
Vehicle	31.8 ± 5.0	66.2 ± 2.1	55.0 ± 4.2	6.3 ± 1.2
JNJ-54717793	26.6 ± 3.2	47.3 ± 5.6^∗^	59.5 ± 2.7	8.6 ± 1.2

*The latency to NREM and REM sleep and the duration of NREM and REM sleep for the first 2 h period after injection (10 mg/kg s.c.) during the light phase are expressed in minutes. Values represent mean ± SEM (*n* = 6). ^∗^*p* < 0.05 versus Vehicle as determined by paired Student’s *t*-test.*

### Transient OX1R Blockade by JNJ-54717793 Selectively Promotes REM Sleep in OX2R Knockout Mice

**FIGURE 3:**
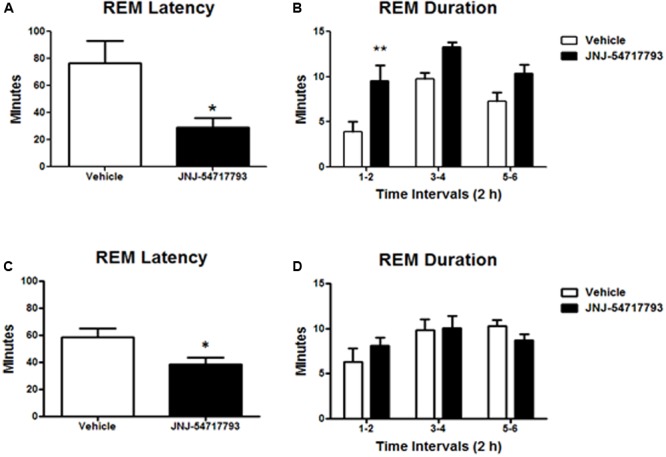
Rapid eye movement (REM) sleep promoting effects of the OX1R antagonist JNJ-54717793 in OX2R KO mice. REM sleep latency and duration in OX2 KO mice **(A,B)** and wild-type **(C,D)** for the first 6 h period after oral dosing (30 mg/kg) during the light phase are expressed in minutes. Values represent the mean ± SEM (*n* = 7 KO, *n* = 5 wild-type). ^∗^*p* < 0.05 and ^∗∗^*p* < 0.01 versus Vehicle as determined by paired Student’s *t*-test (latency) or two-way ANOVA followed by Bonferroni *post hoc* test (duration).

Previous studies have demonstrated that OX1R antagonists produce a disinhibition of REM sleep in the presence of OX2R antagonism (Dugovic et al., 2014; Bonaventure et al., 2015b). In the present study, the *in vivo* functional target engagement was investigated in a model of permanent inhibition of OX2R, and the effects of JNJ-5471773 on sleep-wake states were examined in mice lacking the OX2R relative to their corresponding wild-type mice. As compared to vehicle, oral dosing of JNJ-5471773 (30 mg/kg) at 2 h into the light phase to OX2R KO mice significantly reduced the latency for REM sleep and prolonged the time spent in REM sleep during the first 6 h post-treatment (**Figures 3A,B**). In wild-type mice, JNJ-5471773 slightly reduced REM sleep latency but did not alter REM sleep duration (**Figures 3C,D**). NREM sleep latency and duration were not affected in both genotypes (not shown).

### Effect of JNJ-54717793 in a Model of CO_2_ Induced Panic

**FIGURE 4:**
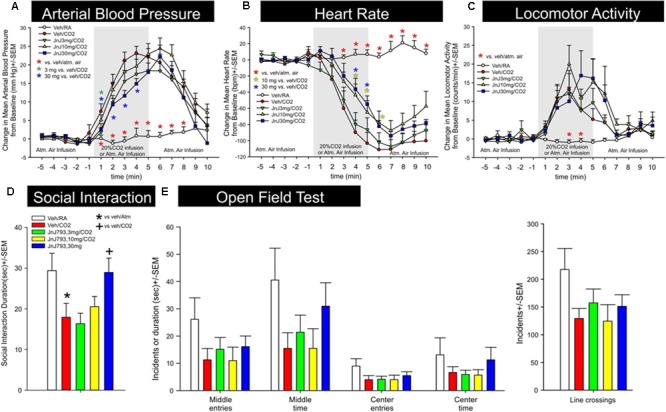
Effect of JNJ-54717793 in a model of CO_2_ induced panic. Line graphs indicate the effects of oral gavage (p.o.) of vehicle, or 3, 10, or 30 mg/kg doses of JNJ-54717793 on 20% CO_2_ induced changes (from 5 min baseline) in **(A)** mean arterial blood pressure (MAP, mm Hg), **(B)** heart rate (HR, beats/min, BPM), and **(C)** general locomotor activity (counts/min) in freely moving rats surgically implanted with radiotelemetry probes. There were no significant differences in baseline mean arterial blood pressure, HR, or locomotor activity (t-5 to t-1). Gray shaded area represents the 5 min CO_2_ or atmospheric air challenge, where atmospheric air (RA) was also infused prior to and after for 5 min (also indicated at bottom of x-axis). ^∗^ Indicates between subjects differences using a Fisher’s LSD post hoc test that was protected with a repeated measures ANOVA and an ANOVA at each time point, p < 0.05. Bar graphs indicate the effects of JNJ-54717793 on 20% CO_2_ induced changes in **(D)** social interaction (SI) in the SI test, and **(E)** line crossings and middle and center time in the open field test. ^∗^ and + respectively indicate between subjects differences between veh/RA and veh/CO_2_ or veh/CO_2_ and 30 mg/kg JNJ-54717793/CO_2_ using a Fisher’s LSD post hoc test that was protected with an ANOVA, p < 0.05.

Activation of the OX1R is a critical component of CO_2_-mediated anxiety and hypertension (Johnson et al., 2012a). Exposing rats to higher concentrations of hypercarbic gas (e.g., 20% CO_2_) depolarizes OX neurons by interacting with pH/ CO_2_ chemosensitive K^+^ channels (Williams et al., 2007), and also causes subsequent release of orexin at post-synaptic targets in the brain and spinal cord to mobilize anxiety-like behavior, hypertension, and increases ventilatory responses (Johnson et al., 2012c, 2015). JNJ-54717793 was tested in a rat model of CO_2_-induced panic. JNJ-54717793 had no effect on baseline cardiovascular activity (see t-5 to t-1 on **Figures 4A,B**). The 30 mg/kg dose of JNJ-54717793 attenuated CO_2_-induced pressor responses at multiple time points (3 mg/kg at one time point) [treatment × time interaction *F*(56,462) = 4.5, *p* < 0.001, *n* = 7,7,8,8,8/group, **Figure 4A**]. The 10 and 30 mg/kg dose of JNJ-54717793 attenuated CO_2_-induced bradycardia responses at multiple time points [treatment × time interaction *F*(56,462) = 8.8, *p* < 0.001, *n* = 7,7,8,8,8/group, **Figure 4B**]. Although JNJ-54717793 had no effect on increases in locomotor activity post CO_2_ (**Figure 4C**), the highest dose attenuated/blocked CO_2_-induced anxiety behavior in the SI test [*F*(3,38) = 3.5, *p* = 0.016, *n* = 7,7,8,8,8, **Figure 4D**] with no apparent sedative effects (line crossings) in the open field test [*F*(4,36) = 1.8, *p* = 0.150, *n* = 7,7,8,8,8, **Figure 4E**]. Nor was there an effect on center or middle times [*F*(4,36) = 0.8, *p* = 0.512, and *F*(4,36) = 1.7, *p* = 0.167, respectively].

### Effect of JNJ-54717793 in a Model of NaLac Induced Panic

**FIGURE 5:**
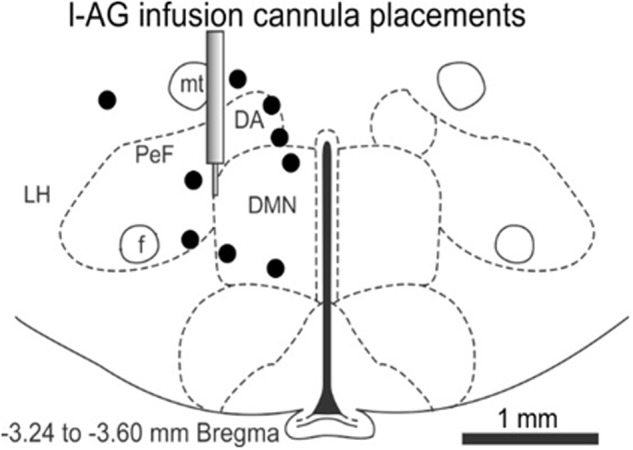
In the 1-allyglycine (l-AG) panic vulnerability model experiment, this illustration shows a representative coronal brain section of the hypothalamus at –3.24 mm from Bregma taken from a Standard Stereotaxic Rat Brain Atlas (Paxinos and Watson, 2005) with circles depicting unilateral cannula placements and infusions of 1-allyglycine (a GABA synthesis inhibitor) that were distributed from –3.24 to –6.70 mm from Bregma and located in the perifornical and dorsomedial hypothalamic region or within ≤0.3 mm from those regions. The illustration of the guide cannula depicts the target region for cannula placements. 1-AG, 1-allyglycine; DA, anterior dorsomedial nucleus of the hypothalamus; DMN, dorsomedial nucleus of the hypothalamus; f, fornix; LH, lateral hypothalamus; mt, mammillothalamic fiber tract; PeF, perifornical hypothalamic area.

The effect of JNJ-54717793 was investigated in a model of panic vulnerability where chronic disinhibition of the perifornical hypothalamic area orexin region leads to exacerbated panic responses to a well-known interoceptive panicogenic challenge (i.e., sodium lactate). The 1-allyglycine infusion cannula placements were all located within the perifornical and dorsomedial hypothalamic area from -3.24 to -3.60 in a distribution consistent with prior 1-allyglycine studies in this panic model (Johnson and Shekhar, 2006; Shekhar et al., 2006, 2011; Johnson et al., 2008, 2010, 2012b) (**Figure 5**), and also in close proximity to orexin neuron distributions that are located from -2.64 to -3.60 mm from Bregma (Peyron et al., 1998).

**FIGURE 6:**
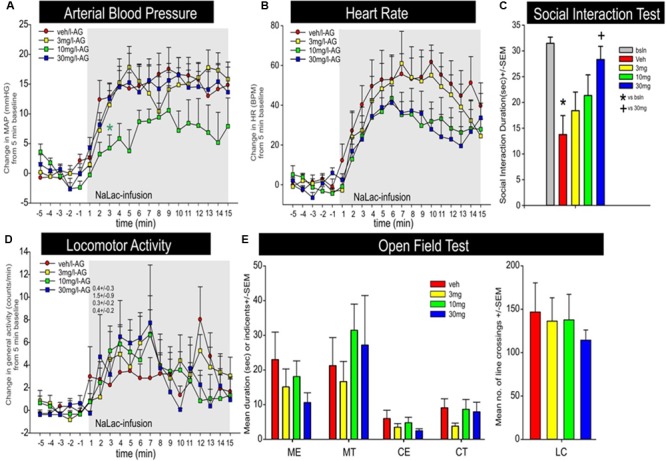
Effect of JNJ-54717793 in a model of sodium lactate induced panic. Line graphs indicate the effects of oral gavage (p.o.) of vehicle, or 3, 10, or 30 mg/kg doses of JNJ-54717793 on intravenous sodium lactate (NaLac) induced changes (from 5 min baseline) in **(A)** mean arterial blood pressure (MAP, mm Hg), **(B)** HR *[beats/min (BPM)]*, and **(D)** general locomotor activity (counts/min) in freely moving rats surgically implanted with radiotelemetry probes. There were no significant differences in baseline MAP, HR, or locomotor activity (t-5 to t-1 minute). Gray shaded area represents the NaLac infusions. ^∗^ *Indicates* between subjects differences using a Fisher’s LSD *post hoc* test that was protected with a repeated measures ANOVA and an ANOVA at each time point, *p* < 0.05. Bar graphs indicate the effects of JNJ-54717793 on NaLac induced changes in **(C)** SI in the SI test, and **(E)** line crossings and middle and center time in the open field test. ^∗^ and + respectively indicate between subjects differences between baseline and veh/NaLac or veh/NaLac and 30 mg/kg JNJ-54717793/NaLac using a Fisher’s LSD *post hoc* test that was protected with an ANOVA, *p* < 0.05.

JNJ-54717793 had no effect on baseline cardiovascular activity (see t-5 to t-1 on **Figures 6A,B**). Although there was no treatment × time or overall treatment effect [*F*(3,29) = 2.8, *p* = 0.090] detected, an individual ANOVA with *post hoc* test at the t3 timepoint revealed an attenuation of pressor response with the 10 mg/kg dose of JNJ-54717793 [*F*(3,32) = 3.3, *p* = 0.034, *n* = 7,8,10,8, **Figure 6A**]. Compared to the vehicle group JNJ-54717793 did not attenuate sodium lactate-induced tachycardia [treatment × time interaction *F*(57,570) = 0.9, *p* = 0.780, *n* = 7,8,10,9, **Figure 6B**]. JNJ-54717793 did not significantly alter locomotion [treatment × time interaction, *F*(57,608) = 0.6, *p* = 0.987, *n* = 8,9,10,9, **Figure 6D**], but the sodium lactate also did not significantly increase locomotion [*F*(3,32) = 0.1, *p* = 0.934]. Although JNJ-54717793 had minimal effects on cardioexcitation, the highest dose did attenuate/block sodium lactate-induced anxiety behavior in the SI test [*F*(4,40) = 5.1, *p* = 0.002, *n* = 8,9,10,9, **Figure 6C**] with no apparent sedative effects (line crossings) in the open field test [*F*(4,54) = 1.9, *p* = 0.128, *n* = 11/group, **Figure 6E**]. Nor was there an effect on center or middle times [*F*(4,54) = 0.9, *p* = 0.490 and *F*(4,54) = 1.2, *p* = 0.340, respectively]. The unequal n’s/per group were due to video malfunctions or complications with radiotelemetry data acquisition.

## Discussion

In the present study we initially determined that JNJ-54717793 is a high affinity, potent and selective OX1R antagonist. After systemic administration JNJ-54717793 crossed the blood brain barrier, occupied OX1Rs in the rat brain and minimally affected spontaneous sleep. We then utilized two different panic models (i.e., threshold panic induction with 20% CO_2_ exposure in normal rats and subthreshold panic induction with intravenous sodium lactate infusion in panic-prone rats), and demonstrated that pretreatment with JNJ-54717793 attenuated CO_2_ and sodium lactate induced panic-like behaviors and cardiovascular responses without altering baseline locomotor or autonomic activity. These data are consistent with previous reports of selective OX1R antagonists attenuating CO_2_ and sodium lactate induced panic-associated behavior and cardiorespiratory responses (Dias et al., 2010; Johnson et al., 2010, 2012d, 2015; Bonaventure et al., 2015b) with little effect on sleep-wake states (Dugovic et al., 2014). In contrast, benzodiazepines are also panicolytic in these models, but do reduce locomotor activity at panicolytic doses (Bonaventure et al., 2015b; Johnson et al., 2015), which is consistent with sedative effects being a significant side effect clinically (Charney and Heninger, 1985; Tesar and Rosenbaum, 1986; Ballenger et al., 1988). In the 20% CO_2_ model, a dual orexin receptor antagonist (i.e., DORA-12) has also been shown to attenuate some aspects of the panic response, but this appears to be primarily through the OX1R and not OX2R since a highly selective and brain penetrant OX2R antagonist (JNJ-10397049) did not alter panic responses to 20% CO_2_ (Bonaventure et al., 2015b; Johnson et al., 2015). In other stress paradigms, both selective OX1R antagonists and selective OX2R antagonists have been shown to have anxiolytic/panicolytic effects. For instance, both OX1R and OX2R contribute to cardiorespiratory responses post-perifornical hypothalamic area disinhibition (Beig et al., 2015b), and both the OX1R antagonist ACT335827 and the selective OX2R antagonist EMPA attenuate novelty stress (cage changes) induced cardioexcitation (Beig et al., 2015a). Yet, like benzodiazepines, selective OX2R antagonists induce and prolong sleep in rodents (Dugovic et al., 2009; Mang et al., 2012; Bonaventure et al., 2015a), thus making them less ideal than selective OX1R antagonists. Collectively, these results and previous data suggest that selective OX1R antagonists may represent a novel approach of treating anxiety disorders, without sedative effects.

Both panic models used here induce significant increases in *ex vivo* cellular c-Fos activity within brain circuits strongly implicated in innate panic (and associated with panic attacks), and in the panic vulnerability model there is also increase in activity within fear associated learning centers (and associated with strong phobia comorbidity with recurrent panic attacks). In the 20% CO_2_ model in naïve rats, panic responses are associated with increases in cellular activity within the perifornical hypothalamic area orexin neurons and cardiorespiratory brainstem circuits (Johnson et al., 2005, 2011, 2012d). In the sodium lactate model, there are additional increases in cellular responses within the amygdala and bed nucleus of the stria terminalis (Johnson et al., 2008). Although orexin neurons innervate many brain regions, they are particularly dense in these brain regions that are mobilized in innate panic and learned fear response (Peyron et al., 1998; Nambu et al., 1999). These include noradrenergic locus coeruleus, and serotonergic systems in midbrain raphe which are current therapeutic targets for treating anxiety disorders, brainstem cardiorespiratory nuclei and bed nucleus of the stria terminalis and amygdala. In many brain regions both OX1R and OX2R receptors are significantly expressed (Trivedi et al., 1998; Marcus et al., 2001). Yet in other regions the expression is more limited to the OX1R or OX2R. For instance, histaminergic neurons in the tuberomamillary nucleus (TMN) have high expression of the OX2R and almost undetectable expression of the OX1R (Eriksson et al., 2001; Marcus et al., 2001). The TMN plays a critical role in wake promotion (Bayer et al., 2001; Huang et al., 2001) and the OX2R on TMN neurons is a major target for wake promoting properties of OX neurons. Conversely, OX1R have a higher expression within the limbic systems (bed nucleus of the stria terminalis and amygdala), cingulate cortex, and noradrenergic locus coeruleus. Accordingly, the functional significance is that OX2R antagonists have potent sleep promoting effects (Bonaventure et al., 2015a; Gotter et al., 2016), which are not apparent with OX1R antagonists (Bonaventure et al., 2015b). Pretreating rats with an OX1R antagonist prior to administering a panicogenic drug (i.e., FG-7142) blocks FG-7142 induced increases in cellular activity in innate panic and learned fear brain circuits that have higher expression of OX1R versus OX2R (e.g., amygdala and dorsal periaqueductal gray and sympathetic nuclei) (Johnson et al., 2012c). Taken together, the panicolytic effects of the OX1R antagonist used here lends further support that OX1R antagonists may be more effective than OX2R antagonists for treating anxiety and fear associated disorders in the absence of sedative effects.

Although the orexin system and OX1Rs appear to be a novel therapeutic target for treating anxiety symptoms/disorders, it is important to note that orexin and orexin receptors may not be involved in all stress responses. For instance, in addition to CO_2_ and sodium lactate challenges, OX neurons are activated during arousal, exploration and footshock stress, but not by restraint or cold exposure stress (Furlong et al., 2009). Furthermore, in these same studies the dual orexin receptor antagonist almorexant attenuated locomotor and cardiovascular responses to exploration and footshock, but not to restraint or cold stress. In mild stress paradigms, OX1R antagonists also do not alter anxiety-associated avoidance behavior in the elevated plus maze test or SI test (Yeoh et al., 2014). Yet, in more aversive stress paradigms, OX1R antagonists do reduce anxiety/fear-associated behaviors in the fear conditioned startle and in resident intruder tests (Steiner et al., 2012, 2013). In other stress paradigms both receptors are involved. Specifically, systemic administration of selective antagonist at OX1R (ACT335827) or at OX2R (EMPA) attenuates novelty stress (cage changes) induced cardioexcitation (Beig et al., 2015a), and both OX1R and OX2R contribute to cardiorespiratory responses post-perifornical hypothalamic area disinhibition (Beig et al., 2015b).

Orexin receptor antagonists have also been implicated in attenuation of fear-associated learning. For instance, in a cue induced fear conditioning paradigm, Sears et al. (2013) reported that OX enhances fear acquisition via OX1Rs and not OX2Rs and through a locus coeruleus-amygdala pathway. This is consistent with a study showing that systemic administration of OX1R, but not OX2R antagonists also attenuate freezing following contextual fear conditioning (Wang et al., 2017), and another study demonstrating that following cue or contextual fear conditioning, systemic or intra-amygdala administration of an OX1R antagonist enhanced extinction, and intracebral infusions of OXA impaired extinction (Flores et al., 2014). Yet, in this study administration of an OX2R antagonist did enhance contextual fear extinction while the orexin-B administration had no effect. Pro fear circuits such as the central amygdala and locus coeruleus are moderately and densely innervated by orexin neurons, respectively (Peyron et al., 1998) with the amygdala expressing more OX1R than OX2R and the locus coeruleus expressing exclusively OX1R (Marcus et al., 2001).

## Conclusion

Although OX1R antagonists may not alleviate mild anxiety or all anxiety associated symptoms they do appear to be effective in rapidly reducing panic and fear associated responses in most stress paradigms, especially as they become more severe, without sedation side effects that would be associated with OX2R antagonists or fast acting panicolytic benzodiazepines (Nutt et al., 2002; Baldwin et al., 2005; Bandelow et al., 2008; Cloos and Ferreira, 2009). OX1R antagonists also have an advantage over SSRIs which become effective 2–3 weeks after daily use (Pollack et al., 2007a,b) and tend to increase anxiety symptoms initially both clinically (Goddard et al., 2001) and preclinically (Ravinder et al., 2013).

## Author Contributions

Participated in research design: PB, PJ, AS, TL, CD; conducted experiments: SY, SF, DN, BL, MW; contributed new reagents or analytic tools: BS, CP, NC; performed data analysis: PB, PJ, DN, BL, CD; wrote or contributed to the writing of the manuscript: PB, PJ, CD.

## Conflict of Interest Statement

The authors have reviewed the journal’s policy and have the following competing interests: PB, CD, BS, SY, CP, BL, DN, NC, and TL are paid employees at Janssen Research & Development, LLC. This does not alter the authors’ adherence to the journal’s policies on sharing data and materials. The other authors declare that the research was conducted in the absence of any commercial or financial relationships that could be construed as a potential conflict of interest.
